# Focus on the Possible Role of Dietary Sodium, Potassium, Phosphate, Magnesium, and Calcium on CKD Progression

**DOI:** 10.3390/jcm10050958

**Published:** 2021-03-01

**Authors:** Sandro Mazzaferro, Natalia de Martini, Jorge Cannata-Andía, Mario Cozzolino, Piergiorgio Messa, Silverio Rotondi, Lida Tartaglione, Marzia Pasquali, on behalf of the ERA-EDTA CKD-MBD Working Group

**Affiliations:** 1 Department of Translational and Precision Medicine, Sapienza University, 00185 Rome, Italy; nataliademartini@virgilio.it (N.d.M.); lidatartaglione@hotmail.it (L.T.); 2 Nephrology Unit Policlinico Umberto I Hospital, 00185 Rome, Italy; marzia.pasquali@uniroma1.it; 3 Bone and Mineral Research Unit, Hospital Universitario Central de Asturias, Instituto de Investigación Sanitaria del Principado de Asturias (ISPA), Retic REDinREN-ISCIII, Oviedo University, 33011 Oviedo, Spain; cannata@hca.es; 4 Department of Health Sciences, Renal Division, University of Milan, San Paolo Hospital, 20122 Milan, Italy; mario.cozzolino@unimi.it; 5 Department of Clinical Sciences and Community, University of Milan, San Paolo Hospital, 20122 Milan, Italy; piergiorgio.messa@policlinico.mi.it; 6 Dialysis Unit at ICOT Hospital, Polo Pontino Sapienza University, 04100 Latina, Italy; silverio.rotondi@libero.it

**Keywords:** sodium, potassium, phosphate, magnesium, calcium, CKD, dietary electrolytes, CKD-MBD

## Abstract

The impressive estimated number of chronic kidney disease (CKD) patients in the world justifies any possible effort at implementing preventive measures of disease progression. Renal insufficiency is associated with significant changes in the electrolyte handling and body balance of sodium, potassium, phosphate, magnesium, and calcium, all of which are biologically vital molecules. Dietary habits could contribute significantly to the optimal management of possible derangements. In this review, we examined the available evidence recommending dietary prescriptions for these five elements aiming at reducing CKD progression. Clear evidence that specific dietary prescriptions may halt or reduce CKD progression is lacking. However, some practical recommendations are possible to prescribe the best possible therapy to the individual CKD patient.

## 1. Introduction

Prevention of chronic renal failure progression is even more essential now that the number of chronic kidney disease (CKD) patients is estimated to reach 850 million in the world [1]. From the perspective of prevention, the role of dietary habits could be relevant and in need of better appreciation to improve the management of well-established modifiable risk factors of CKD progression like blood pressure, proteinuria, phosphate [2], and lithiasis [3]. Among electrolyte derangements, besides sodium, commonly associated with body volume and blood pressure, a modified attention to serum levels of potassium is emerging, suggesting a modification of the traditional generic dietary restriction and careful discontinuation of Angiotensin Converting Enzyme-inhibitors to avoid potassium increments that could be more dangerous than mild-moderate hyperkalemia in chronic renal failure (CRF) patients [4]. Similarly, derangements in mineral metabolism (e.g., serum calcium and phosphate) are even more associated with hard cardiovascular outcomes, but also with bone disease and bone fractures, which are now recognized as risk factors of morbidity and mortality [5], claiming for novel appreciation with invasive and non-invasive diagnostic tools like bone biopsy [6], vertebral morphometry [7], Magnetic Resonance Imaging (MRI) [8], and Positron Emission Tomography (PET) [9]. In fact, the disorders of bone and minerals occurring early in renal failure might represent an interesting biologic model of accelerated aging [10] and of the associated progressive reduction of the glomerular filtration rate (GFR). In this scenario, a disturbed crosstalk between bone and kidney implicates subtle changes in the composition of body fluids and electrolytes, and dietary mineral intakes become relevant for the control of five elements in particular, that is, sodium, potassium, phosphate, magnesium, and calcium, all of which exert vital biological roles and need to be adequately supplied in CKD.

International scientific societies periodically update the recommended dietary intake of minerals that cover the requirements of the general population. In CKD, these four minerals require specific dietetic advice to avoid either insufficient supply or possible accumulation. This review explores the recommended intakes and beneficial or dangerous effects of sodium, potassium, phosphate, magnesium, and calcium in healthy and CKD populations, aiming at underlying potential benefits for CKD progression.

First of all, because terminology is highly variable, we selected some terms to be used in the present article: adequate intake (the observed or experimentally derived intake that appears to sustain health in a defined population); recommended dietary allowance (the intake that meets the needs of 97 to 98% of healthy individuals in a particular age- and sex-specific group), which is mostly used in United States and Canada and is similar to the reference nutrient intake used by the World Health Organization (WHO)/Food and Agricultural Organization (FAO); and tolerable upper intake level (the highest daily intake associated with no risk of adverse effects on health) [11].

## 2. Sodium

There are some 55 to 65 mmol/kg (1.3 to 1.5 g/kg) of body weight of sodium in the body, mostly contained in the extracellular fluids (95%) and with blood levels averaging 135 to 145 mmol/L. Intestinal absorption from foods and fluids is almost complete and excessive amounts are excreted by the kidneys [12]. Because some 99% of the filtered sodium is reabsorbed by tubular cells, the role of the kidneys in sodium balance is relevant. Sodium is naturally present in water and foods and is widely employed to preserve or season foods. Dietary intake is best estimated through 24 h urine excretion as dietary recalls invariably underestimate assumptions [13]. Worldwide daily intakes range between 95 mmol (2.18 g) and 240 mmol (5.51 g), corresponding to roughly 5 to 13 g of sodium chloride, with an average consumption of 172 mmol/day (3.95 g/day), i.e., about 10 g of sodium chloride. Dietary habits significantly affect these values, which are lowest in sub-Saharan Africa and highest in Asian countries [14,15,16]. According to the National Academies of Sciences Engineering and Medicine, there is insufficient evidence to establish precise requirements of dietary sodium, however, a causal and intake–response relationship is evident between sodium intake and chronic disease risk [17]. Therefore, according to WHO, dietary intake should be limited to less than 87 mmol/day (2.0 g/day) (Table 1), which corresponds to less than 5 g of sodium chloride, as this is expected to improve blood pressure control and the eventual cardiovascular risk [18]. In renal patients, in the absence of specific tubular defects, derangements in blood levels are rare and mostly limited to the end stage phase of the disease. Accordingly, dietary intake recommendation should be similar to the general population, except in the case of tubular defects requiring supplements. The therapeutic target when evaluating or prescribing sodium intake in renal patients is thus represented by 24 h urine excretion and blood pressure and body fluids control. As indicated by the observed positive relationship between urinary sodium excretion and CKD progression [19], positive effects are possible through improvement of blood pressure and volume control. Nonetheless, the recently described positive effects of Sodium Glucose co-Transporter 2 (SGLT2) inhibitors on CRF progression in non-diabetic patients suggest that direct effects are possible through actions on tubulo-glomerular feedback [20]. A final consideration could be that urinary sodium is positively associated with calcium excretion, and that excessive dietary loads may result in hypercalciuria and eventual renal risks. In summary, the commonly recommended lifestyle modification suggestion to prefer fresh foods and to avoid salt seasoning can be directly translated to renal patients.

## 3. Potassium

The human body contains about 3500 mmol of potassium, which is mostly stored in the intracellular fluid (98%) and circulates in blood at concentrations between 3.5 and 5.0 mmol/L. Excretion of excessively absorbed potassium is mostly renal (80%) and, in minor part, intestinal, in normal conditions. The main food sources are from unprocessed fruits and vegetables and high intake is safe as healthy individuals excrete excess potassium in the urine with no increment in blood levels [21]. Dietary potassium intake is best estimated by 24 h urine collection, but less reliable methods like spot urine sampling or dietary recall and food frequency questionnaire (FFQ) are also used. Estimated average intakes in different countries range from 52 mmol/day in China to 68 mmol/day in the Unites States and 83 mmol/day in Europe [22,23,24]. However, each of these dietary potassium intakes is lower than the >90 mmol/day (>3.5 g/day) recommended by the WHO in adults (Table 1). This recommendation arises from the available evidence that higher potassium intake has beneficial effects on blood pressure control. In fact, high potassium diets reduce blood pressure in hypertensive patients, improve its control in normotensives, and reduce the risk of stroke; on the contrary, low potassium diets (<40 mmol/day or 1.5 g/day) are associated with higher blood pressure and adverse cerebral events [21]. Following the acknowledgement of these inverse associations, a role for the urinary sodium–potassium ratio (UNa/K) has been suggested in the management of blood pressure [25]. This ratio immediately highlights a common dietary habit imbalance (high sodium and low potassium), is not influenced by the quality of urine collection, and should be ideally close to 1 [26].

The mechanisms linking potassium intake and blood pressure are not well understood. There is some evidence that low dietary potassium activates thiazide-sensitive NaCl cotransporter (NCC) in renal tubules, thus causing sodium retention, while, on the contrary, high potassium diets decrease NCC levels and promote natriuresis, through a plasma potassium dependent effect that modulates the voltage of cell membranes in the distal convoluted tubule [27]. Accordingly, the effect of dietary potassium on blood pressure control could be through natriuresis.

Differently from the general population, renal patients frequently experience serum potassium derangements, characterized by a similar prevalence of hyperkaliemia (15–20%) and hypokalemia (15–18%), both associated with similar rates of adverse outcomes and mortality. For this reason, inadequate dietary potassium intake is an issue in CKD patients [28]. Recently, the 2020 Kidney Disease Improving Global Outcome (KDIGO) Controversies Conference concluded that the association between dietary intake and serum levels of potassium is weak in CKD and that there is no reason to generically adopt the common policy of restricting dietary potassium in renal patients. Moreover, evidence is lacking that dietary restriction or liberalization has beneficial effects in CKD. Therefore, dietary potassium intake should be tailored to the different CKD stages and to the individual response of patients, having the following reference intake values: at least 104 mmol/day for CKD stages G1–2, 54–104 mmol/day in CKD stages G3–4, 78–104 mmol/day for peritoneal dialysis patients, and 70–78 mmol/day in hemodialysis [29] (Table 1).

As for CKD progression, specific data on the role of dietary potassium are not invariably consistent. Low urinary potassium excretion is associated with an increased risk of developing CKD in the general population [30]. High 24 h urinary potassium lowered the risk of GFR decline with no risk of hyperkaliemia in stage G2 CKD patients [31] and reduced the risk of GFR decline or end-stage renal disease (ESRD) in CKD stage 2–3 patients [32]. However, in another study, the relationship between high urinary potassium and the risk of eGFR drop was positive [19]. As for the UNa/K ratio, it was recently reported to be positively associated with blood pressure values and with uncontrolled or treatment-resistant hypertension in patients with moderate to severe CKD, similarly to UNa/creatinine [33]. 

A final additional issue to consider with potassium is food quality, as we know that a plant-based diet might be beneficial in patients with CKD. Indeed, in CKD-stage G3, a fruit- and vegetable-rich diet or the addition of oral bicarbonate in the diet improved albuminuria and CKD progression, as compared with usual care, and this beneficial effect could be referred to reduced acid load and to higher potassium and alkali supply in the plant-based diet [34]. In addition, as changes in urine composition represent specific aspects in the management of patients with nephrolithiasis in whom an increased potassium excretion is commonly recommended, food quality represents a significant possible source. On the contrary, a reduction of dietary potassium load can be obtained, if necessary, by boiling foods. This could be specifically helpful in patients suffering constipation, as it is known to favor intestinal absorption.

In summary, the role of dietary potassium in the progression of CKD is not settled. However, increased attention to urinary potassium excretion and to food quality could be helpful to prescribe, in the individual patient, the dietary amount that allows optimal serum levels and is associated with the best possible blood pressure and acid–base control. Further studies should investigate the effect of dietary restriction in CKD on potassium circulating levels, the effect of fruit- and vegetable-rich diets in CKD, the impact of dietary potassium restriction in people with CKD on clinically important outcomes, and the effects of unrestricted fruit/vegetable intake on the risk of hyperkalemia in advanced CKD or dialysis.

## 4. Phosphate

Phosphate in the human body averages 26,600 mmol (825 g), and is mainly stored in bone (85%) and soft tissues (14%), with only 1% circulating in blood, where normal levels range between 0.81 and 1.45 mmol/L. Normal blood levels mainly result from renal excretion.

Phosphate-rich foods are dairy products, meat, eggs, fish and grain. Phosphate from legumes has low bioavailability, while phosphate added to foods as preservatives is highly absorbable and may contribute substantially to total phosphate intake (up to 10–30%). Excessive intake, e.g., from phosphate laxatives, may produce acute adverse effects like hyperphosphatemia and hypocalcemia, acute interstitial nephritis, and other severe systemic effects in healthy subjects.

Similarly to potassium, the reference test to estimate dietary intake is 24 h urine concentration, but FFQ and dietary recall are also employed. The Institute of Medicine Food and Nutrition Board indicated 23 mmol/day (700 mg/day) as the phosphate recommended dietary allowance in the adult general population, either for males or females, which is the same value for any stage of CKD. Moreover, given the potential detrimental effects of excessive phosphate intakes, an upper intake level of 130 mmol/day is defined (Table 1) [35,36]. Regrettably, the estimated average intakes in Western countries are much higher than the recommended dietary allowance (58 and 43 mmol/day in U.S. males and females respectively, and 52 mmol/day in Europe), and data from NHANESIII cohort highlighted that phosphate consumption above 45 mmol/day (1400 mg/day) was associated with increased mortality rate in healthy individuals [37]. In non-dialysis-CKD patients, a clear association is evident between serum phosphate levels and CKD progression [38], which would suggest that lowering serum phosphate by dietary restriction might reduce serum levels and the probability of developing ESRD. However, in CKD patients, the relationship between serum phosphate levels and dietary phosphate intake as evaluated by 24 h urinary collection is not tight and restriction is expected to mainly affect renal fractional excretion. Selamet et al. [39] retrospectively examined data from the Modification of Diet in Renal Disease (MDRD) study and observed that 24 h urinary phosphate excretion had only a modest inverse correlation with serum phosphate concentration, and no association with the risk of ESRD or all-cause mortality. By contrast, experimental studies have shown that a high phosphorus diet not only increases vascular calcification and decreases bone mass, but also reduces glomerular filtration rate and increases mortality [40,41]. Further, no randomized controlled trial is available to demonstrate that reducing dietary phosphate intake improves cardiovascular and renal outcomes in CKD, and the most recent KDIGO guidelines recommend restriction only to treat hyperphosphatemia [42].

However, new discoveries could come from a better understanding of the cross talk between bone and kidney, described by a number of new bone biomarkers [43] and in particular by the novel phosphaturic hormone fibroblast growth factor 23 (FGF23) [44], whose levels increase early in CRF as a response of bone cells to the somehow perceived phosphate load. In animal models of CKD, the phosphaturic load per single nephron resulting from FGF23 increments causes tubular and interstitial damage [45], thus amplifying nephron loss [46]. Accordingly, by limiting FGF23 increments with dietary phosphate restriction, beneficial effects are possible in CKD progression [47]. In a clinical study, switching 99 CKD stage G3–4 patients from a low protein diet to a very low protein diet plus chetoanalogues produced a significant reduction in dietary phosphate associated with a drop in serum and urinary phosphate and the halving of proteinuria [48], thus pointing to a role for phosphate in CKD progression [2].

Further, dietary phosphate sources need to be considered. In fact, in CKD stage G3–4 patients receiving a vegetarian diet, serum levels and fractional excretion of phosphate were lower than when receiving a meat diet with the same supply of proteins and phosphate [49]. Most probably, the greater quantity of fibers in the former diet reduced the intestinal absorption of phosphate and the eventual renal phosphate load. Moreover, the quality of food and renal excretion of phosphate are part of the dietary counselling and preventive management of nephrolithiasis, requiring specific evaluation according to the different type of stone disease. Cooking methods, like boiling, represent a further helpful way of reducing dietary phosphate load [50].

In summary, as renal phosphate load represents a potential factor of renal damage, controlling its intake seems prudent in the general and CKD populations. Food quality choice and dietary phosphate load assessed with urinary collection represent helpful tools to detect and avoid otherwise unnoticeable and undesirable excesses with potential detrimental effect on CKD progression.

## 5. Magnesium

The human body contains about 823 mmol (20 g) of magnesium, distributed among the skeleton (65%), intracellular space (33%), and extracellular space (2%). Normal blood levels range between 0.65 and 1.05 mmol/L [51]. Magnesium balance mainly involves intestinal (67%) and renal (33%) handling and, therefore, gastrointestinal or renal disease are the clinical conditions most frequently associated with serum derangements.

The main dietary sources are nuts, grains, fish, vegetables, and legumes. High intakes are generally compensated by increased urinary excretion, and excessive amounts can cause diarrhea or gastrointestinal symptoms or, rarely, in case of hypermagnesemia, more severe clinical toxicity (hypocalcaemia, hypotension, bradycardia, muscle paralysis, and so on).

Magnesium intake can be estimated with 24 h urine collection or with dietary recalls. However, because no specific hormone exists for magnesium metabolism and the principal site of storage, which is bone, does not react promptly to dietary shifts, serum levels roughly reflect the average and ongoing food intake.

According to WHO, the reference nutrient intake for magnesium varies with age and gender and should be at least 10.8 mmol/day (260 mg/day) in adult males and 9.2 mmol/day (220 mg/day) in females, while the upper intake level is set at 14.6 mmol/day (350 mg/day) (Table 1) [52]. More recently, the European Food Safety Authority indicated higher values of adequate intake (14.6 mmol/day or 350 mg/day in males and 12.5 mmol/day or 300 mg/day in females) [53]. In any case, all of these references are higher than the estimated average assumed by the general populations in China (8 mmol/day), in Europe (9.6–13 mmol/day), or in United States (males 13 and females 8.6 mmol/day) [52]. The reason there is a tendency to increase the value of adequate intake is that it is expected to contribute to avoidance of low levels of serum magnesium, which have been associated with higher vascular risk or disease (endothelial dysfunction; wall calcification; and inflammatory, atherogenic, and pro-thrombotic responses). Moreover, oral supplements could lower blood pressure values [54] even though the evidence linking dietary magnesium and blood pressure is still inconsistent [51].

There are few data dealing with the role of dietary magnesium and CKD progression. For example, in the Atherosclerosis Risk in Communities (ARIC) study, including non-CKD participants with eGFR > 60 mL/min/1.73 m^2^, higher dietary magnesium evaluated through FFQ was associated with a lower risk of CKD [55]. Similarly, from this study, but in the larger population with available serum magnesium assay, those with lower levels had a significantly higher risk of developing CKD or ESRD [56]. In another study, in an urban population of 1252 participants with baseline GFR higher than 60 mL/min, magnesium intake was evaluated through 24 h dietary recalls and the lowest tertile of dietary magnesium intake showed twofold greater odds of developing rapid kidney function decline [57]. In hemodialysis patients, who are mainly prone to hypermagnesemia, higher magnesium levels have been shown to mitigate the cardiovascular risk induced by hyperphosphatemia [58]. No evidence is available in patients on conservative therapy for renal insufficiency. Actually, there are no suggested intakes for magnesium in CKD population (Table 1).

Similarly to previous ions, the type of food needs consideration. Plant-based diets, beyond producing alkalinization, phosphate load reduction, urinary potassium increments, gut microbiota, and intestinal transit improvements, are expected to increase magnesium availability and serum levels [59]. In addition, given the undisputed role of magnesium in inhibiting the process of calcification, a preventive therapeutic role is commonly recognized in patients with nephrolithiasis. Therefore, vegetarian diets providing higher availability of magnesium could have potential beneficial effects. As a whole, however, the role of dietary magnesium in the progression of CKD, although attractive, is not adequately investigated and represents a field in need of clinical research with Randomized Clinical Trials

## 6. Calcium

Calcium content in the body averages 25,000–32,500 mmol (1000–1300 g) and is mostly stored in the skeleton. Less than 1% circulates in blood, where normal levels range between 2.2 and 2.6 mmol/L. Calcium balance results from a complex endocrine regulation of intestinal absorption, renal excretion, and bone buffering.

Food sources of calcium are mainly represented by dairy products, followed by vegetables, legumes, nuts, cereal products, and fortified foods, with some variable contribution from tap or mineral waters. Excessive intake may cause hypercalcemia, hypercalciuria, renal failure, vascular and soft tissue calcification, and nephrolithiasis. On the contrary, inadequate intake results in osteopenia, osteoporosis, and eventual fractures. Daily intake of calcium can be evaluated with FFQ or food recalls only, as urine excretion is subject to very active renal handling and to significant reduction along with GFR decline.

Adequate intake is critical for calcium balance and changes during skeletal growth in children and during bone remodeling in adult ages. For this reason, different recommended dietary allowances are established according to age. Because of the link between dietary calcium, bone health, and risk of fractures, several national evaluations and recommendations are available. The Institute of Medicine Committee in North America recommends 32.5 mmol/day (1300 mg/day) in growing children, 25 mmol/day (1000 mg/day) in adult males and females, and 30 mmol/day (1200 mg/day) in ageing people, while the upper intake level is set in the range between 50 mmol/day (2000 mg/day) and 75 mmol/day (3000 mg/day) (Table 1) [60]. National surveys in the general population indicated adequate calcium intake in the United States and Canada [60], and trends towards insufficient amounts along with age in Europe [22]. Higher calcium intake has been associated with positive effects on hypertension, obesity, and blood lipids, with possible eventual cardiovascular effects. However, the results of intervention trials are controversial [61].

The link between dietary calcium and CKD progression seems marginal except that, if responsible for hypercalcemia/hypercalciuria, it could favour stone formation, hypertension, and renal failure. Dietary intakes may affect serum levels and the resulting low or high serum levels are known to be associated with increased mortality [62,63,64] and CKD progression [65]. In any case, the main concern in CRF patients is excessive intake and calcium load due to the risk of calcifications and negative cardiovascular effects [66].

Calcium intake in CKD should average 20–25 mmol/day (800–1000 mg/day) (Table 1) [67,68], but calcium-based phosphate binders represent an additional confounding source in these patients [69]. In adults on dialysis [70] and in children and adolescents with CKD stage 4–5 D [71], calcium intake has been estimated to be lower than the recommended dietary allowances. As KDIGO guidelines suggest to tolerate mild hypocalcaemia and to limit calcium-based phosphate binders to avoid hypercalcemia [42], low calcium intake is possible, which, however, carries the risk of worsening bone disease and increased fracture [72]. In particular, when a low protein diet is prescribed, a low calcium content is expected, which requires oral calcium salt supplements (commonly between 500 and 1500 mg/day), with either potentially favorable effects on phosphate absorption or undesirable positive calcium balance. Up to now, no randomized controlled trial is available to establish the optimal level of calcium intake in CKD in conservative therapy.

In summary, calcium intake affects calcium balance in CKD, however, the complex endocrine regulation prevents a precise assessment of the final body distribution. The role on CKD progression is secondary to the final effects in terms of serum and urinary levels. 

## 7. Conclusions

In conclusion, the role of dietary intakes of sodium, potassium, phosphate, magnesium, and calcium on the progression of CKD, although hypothetically possible, is still speculative. Indirect effects are possible if modifications are produced on some of the renowned factors of CKD progression (Table 2). We know that blood derangements in particular of potassium, phosphate, magnesium, and calcium induced by dietary challenges in experimental animal models produce renal damage and CKD progression. In addition, observational studies describe associations between serum levels of these four electrolytes and CKD progression, which suggests a possible role for diet. However, the association between dietary load and serum level is not tight in renal patients, and dietary modifications do not improve CKD progression, unless significant serum derangements are produced. On practical grounds, compared with a standard Western diet, vegetarian foods might offer metabolic advantages in CRF. Therefore, generic restrictions of dietary habits might be no more sufficient in CKD patients. Blood assays should be integrated with urinary exams and food questionnaires in order to tailor dietary prescription to each patient according to biochemical and clinical needs.

## Figures and Tables

**Table 1:** Chemical characteristics; body content; and dietary intakes of sodium, potassium, phosphate, magnesium, and calcium.

	Sodium	Potassium	Phosphorus *	Magnesium	Calcium
Symbol	Na	K	P	Mg	Ca
Atomic number	11	19	15	12	20
Molecular weight, g/mol	23.0	39.0	30.9	24.3	40.0
Body content, mmol	3700–4200	3500	26,600	823	25,000–32,500
Intracellular space, %	5	98	14	33	<1
Extracellular space, %	95	2	1	2	<1
Skeleton, %	–	–	85	65	99
Blood levels, mmol/L	135–145	3.5–5.0	0.81–1.45	0.7–1.1	2.2–2.6
Recommended intake °, mmol/day (M/F) ^§^	<87	90/90	23/23	10.8/9.2	25/25
Upper intake level °, mmol/day ^#^	–	–	130	14.6	62.5
Estimated average intake °, mmol/day ^+^					
China, M/F	270/240	52	33/29	8.7/13.7	9.4/8.7
United States, M/F	180/130	68	58/43	13.5/8.5	26
Europe, M/F	170	83	52	14.2/10.6	25/21
Suggested intake in CKD, mmol/day ^^^	<87	52–104	23	?	20–25

* Phosphorus is extremely reactive; thus, it only exists in nature bound to other elements. The most important compound in human biology is phosphate (molecular formula: PO_4_^3−^; molecular weight: 94.9 g/mol). Body content and dietary intakes refer to phosphate. ° referred to adult general population. ^§^ mg/day (M/F): sodium <2000/<2000; potassium 3500/3500; phosphate 700/700; magnesium 260/220; calcium 1000/1000. ^#^ mg/day: phosphate 4000; magnesium 355; calcium 2500. ^+^ mg/day China, United States, Europe (M/F): sodium 6.2/5.5, 4.2/3.0, 3.9; potassium 2000, 2650, 3200; phosphate 1000/900, 1800/1300, 1600; magnesium 212/333, 328/206, 345/258; calcium 376/348, 1040, 1000/840. ^^^ mg/day: sodium <2000; potassium 2000–4000; phosphate 700; calcium 800–1000. CKD, chronic kidney disease.

**Table 2:** Possible effects of dietary electrolyte intakes on some risk factors of CKD progression.

	Sodium	Potassium	Phosphate	Magnesium	Calcium
Blood pressure	+	+	+/−	+/−	+/−
Proteinuria	+/−	+	+	+/−	−
Serum phosphate	−	−	+	+/−	+
Lithiasis	+	+	+/−	+	+

+ plausible; +/− possible; − no evidence.

## Data Availability

No personal data were included in the present narrative review.
